# 17^th^ Congress of the Academy For Multidisciplinary Neurotraumatology: 11-12 April 2019 | Platinia Conference Hall | Cluj-Napoca | Romania

**DOI:** 10.25122/jml-2019-1013

**Published:** 2019

**Authors:** Dragos Cretoiu, Livia Livint Popa

**Affiliations:** 1. Carol Davila University of Medicine and Pharmacy, Bucharest, Romania; 2. Iuliu Hatiganu University of Medicine and Pharmacy, Cluj-Napoca, Romania

The 2019 Congress of the Academy for Multidisciplinary Neurotraumatology took place between April 11-12 in Cluj-Napoca, Romania, organized by the **Academy for Multidisciplinary Neurotraumatology (AMN)**, the **Foundation of the Society for the Study of Neuroprotection and Neuroplasticity (SSNN)**, the **Journal of Medicine and Life (JML)** and partners.

This educational event brought together specialists from multiple related domains of research and clinical practice – neurology, neurosurgery, psychology, neurorehabilitation, neuroproteomics, biomarkers study, and others – who shared their vast experience in neurotraumatology and related fields in various presentations and workshops.

The event set the stage for two days of intensive talks and debates between 150 participants from 7 countries, on a broad range of problems in clinical neurotraumatology and neuroscience, promoting the integration of new scientific information via keynote lectures, teaching-oriented workshops, and round table discussions. A rich and diverse audience of healthcare professionals interested in this steadily expanding and multidisciplinary field attended the event: *medical doctors, patients, physicians, nurses, therapists, public health experts, and clinical researchers*.

Exciting lectures on a diverse set of topics were covered in five presentation sessions, one round-table discussion, and five interactive group workshops.

**Figure 1:**
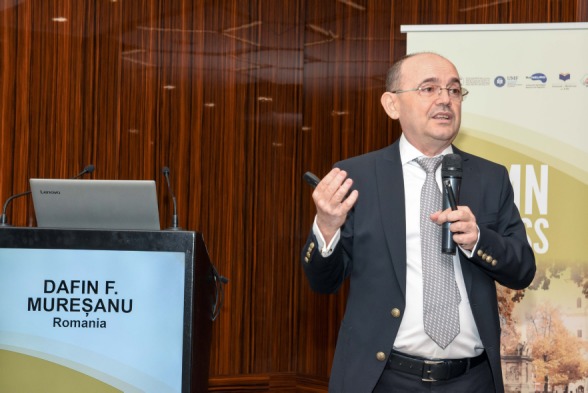
Professor Dafin Muresanu (Romania), Secretary General of AMN, delivering results from the CAPTAIN II Trial.

In the opening, Professor Dafin F. Mureşanu from Romania, AMN’s Secretary-General, President of the European Federation of Neurorehabilitation Societies (EFNR), provided first-glimpse results from the CAPTAIN II study in traumatic brain injury (TBI). This is the first clinical trial that used a multidimensional approach to capture the complexity of TBI health states. In addition to methodological innovations, the presentation emphasized the importance of fostering neurorecovery as an endogenous continuous brain defense response that is anti-correlated with neuroprotection (Figure 1).

**Figure 2:**
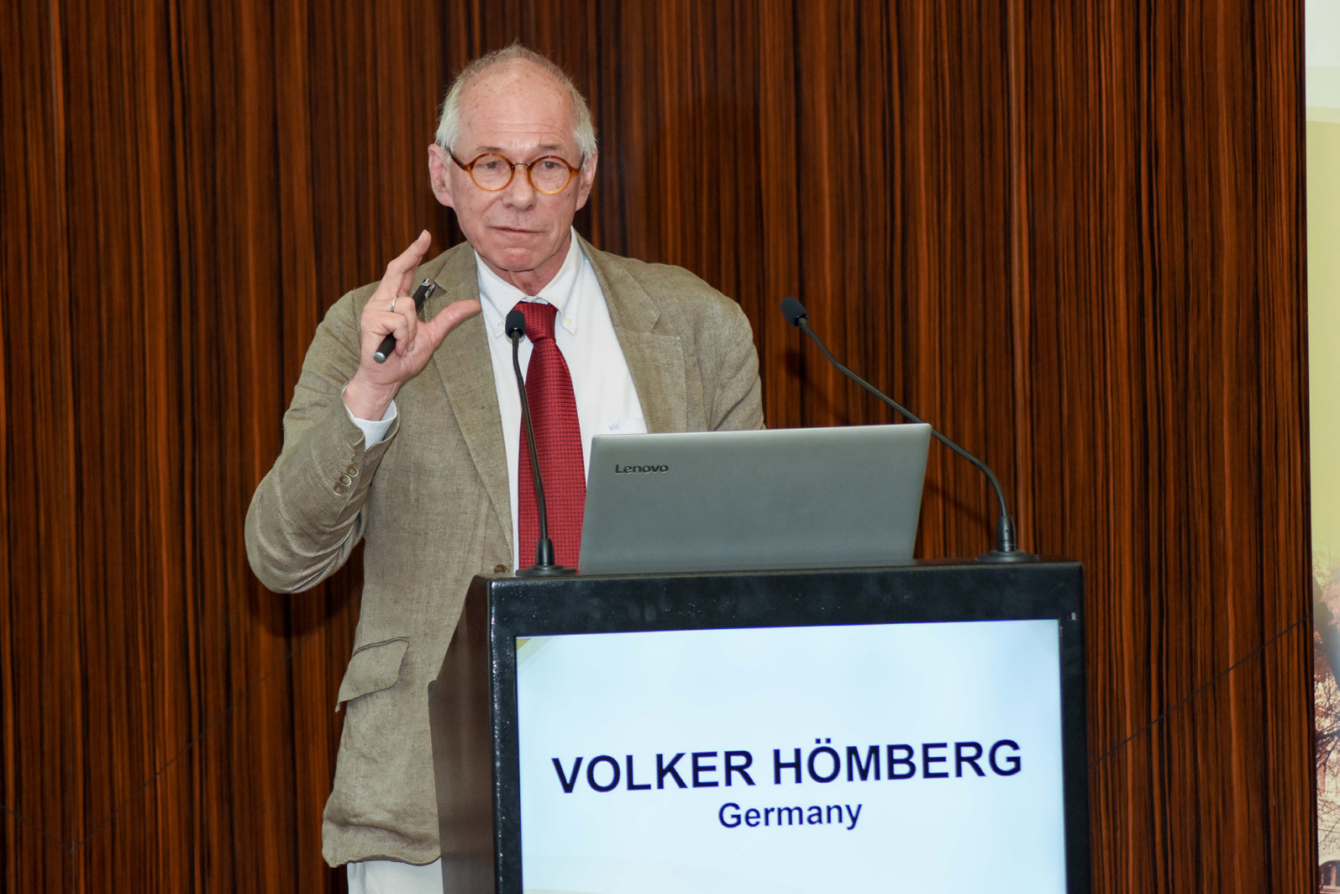
Professor Volker Homberg (Germany), Chairman of the Scientific Program Committee of AMN, discussing strategies to address impairment after neurotrauma.

Professor Volker Homberg (Germany), Chairman of the AMN Scientific Program Committee, Secretary-General of EFNR, and WFNR, further invited the audience to reflect on a difficult conundrum for clinical research and practice: “*Are we really able to influence impairment?*”. Despite research in the field having proven that spontaneous recovery after stroke and TBI is usually 70%, the real challenge is to find advanced and integrated care interventions in order to increase this mark (Figure 2).

To date, the following major strategies showed results to help decrease impairment in the subacute stages: forced use or constraint-induced movement therapy, pharmacological agents (antidepressants – fluoxetine, citalopram – and, more recently, in the CAPTAIN trial, cerebrolysin), neuromodulatory techniques – peripheral neuromuscular and/or sensory stimulation (e.g., whole hand subliminal “mesh-glove” stimulation) and non-invasive brain stimulation techniques, such as repetitive transcranial magnetic stimulation (rTMS) and transcranial direct current stimulation (tDCS).

**Figure 3:**
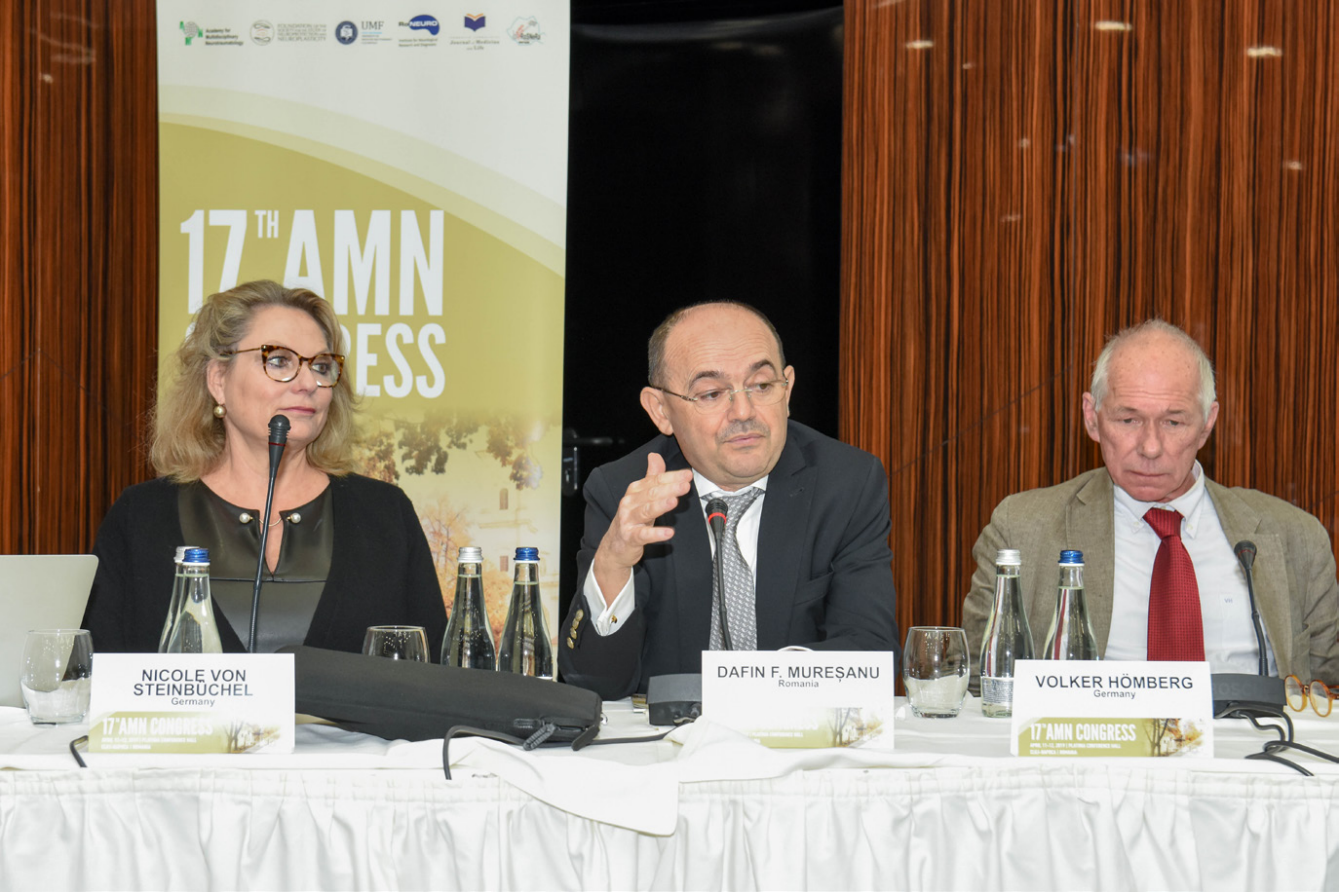
AMN 2019 panel discussion chaired by Professor Nicole von Steinbüchel (Germany) on patient-reported outcomes.

After discussing therapeutic options in neurotraumatology, recent advancements in the field were presented from the patients’ perspective. Professor Nicole von Steinbüchel (Germany), AMN Past President, brought to light a model for patient-reported and performance-based outcomes after TBI from CENTER-TBI, a large, prospective, observational European study that includes a pragmatic data collection of all patients after traumatic brain injury (TBI) (Figure 3). The necessity of the multidimensional approach in TBI was highlighted by Professor Johannes Vester, AMN President, and detailed in the second session by Professors Max Hilz (Germany) and Karin Diserens (Switzerland).

Further on, Professor Christian Matula from Austria revealed the current challenges of neurosurgery – “*Why, where and how to operate?*” – a combination of interaction, precision, and vision. His lecture was followed by Professor Heinrich Binder’s exposition of prolonged disturbances of consciousness after brain injury.

The third session began with a topic addressed to young neurosurgeons and neurologists by dr. Martin Rakusa from Slovenia: *Education in Neurotraumatology*. It was continued by Professor Ştefan Florian’s speech on cranioplasty and Professor Alexandru Ciurea’s research on TBI as a trigger or aggravating factor in Alzheimer’s disease.

In the last session of the day, Professor Yuri Alekseenko from Belarus discussed the diagnosis and initial management of mild traumatic brain injury, Professor Gelu Onose from Romania presented a synthetic up-to-date literature review on the main traumatic spinal cord injury current issues, while Professor Mihaela Băciuţ (Romania) discussed the situation of the 12% of the patients presenting in emergency departments with the coexistence of maxillofacial trauma and TBI. Dr. Dana Boering closed the fourth scientific session with an exciting talk on the early rehabilitation of severe brain injury.

The second day started with a round table discussion on the global burden of TBI, centered on the status-quo assessment of the public health problem. Delegates from Austria, Egypt, Philippines, South Korea, Poland, Russia, Ukraine, Uzbekistan, and Romania provided rich insight and national-level information. The conclusion of the discussion revolved around the potential of using AMN expertise to bridge geographical differences in the treatment and human resource availability in TBI through multidisciplinary, cross-country collaboration.

Professor Jongmin Lee from South Korea sensitized the audience about the profound impacts of severe acquired brain injury on alertness and cognition, with devastating effects on the quality of life of patients. He also reminded the emotional and financial consequences of their families and caregivers. Afterward, he presented the differential diagnostic of disorders of consciousness ranging from a comatose state or vegetative state to a minimally conscious state and different pharmacological or nonpharmacological treatments that could be used for consciousness recovery: amantadine and zolpidem, apomorphine, levodopa and methylphenidate, deep brain stimulation, transcranial direct current stimulation, and multimodal sensory stimulation. Professor Lee particularly spoke about the combined therapy of cerebrolysin and amantadine as a promising treatment option in these cases.

The next presentation, held by Dr. Lynne Lourdes Lucena from the Philippines, was a multi-center, retrospective cohort study about the efficacy of neurotrophic factors in severe traumatic brain injury.

The extensive research concluded that neurotrophic factors are beneficial for severe TBI patients with non-operative lesions: a greater improvement in the Glasgow Coma Scale (GCS) and Glasgow Outcome Score (GOS) and shorter length of hospital stay was evidenced in comparison to standard treatment alone.

The outcome prediction in TBI was discussed by Professor Peter Lackner (Austria). He explained that after the trauma, many factors are influencing the further course of the disease. Hence not only the initial clinical presentation, secondary brain injury, or systemic complications during the acute phase of TBI but also timing and intensity of neurorehabilitation are known determinants of the outcome. Due to patient heterogeneity, large cohorts are needed to identify solid outcome predictors. The international initiative for traumatic brain injury research supports the further development of common data elements for TBI as a method for international comparative effectiveness research (CER). 

The future perspectives were intensively debated in five workshops focused on different topics. Group workshops were essential highlights of the conference, bringing together top-tier specialists on five key topics. First, an international panel of participants tackled the critical problems that *low- and middle-income countries face regarding TBI patient treatment*. Delegates identified the following items of importance: incorrect assessment of the TBI, insufficient number of neurosurgeons, treatment availability, competencies for acute, post-acute and long-term staff, and precarious curriculum of the medical specialization. The requirement of the rapid transport from the place of accident to the emergency hospital and the need for acute units of neurorehabilitation integrated into acute hospitals were highlighted as key care standards. 

Concerning acute neurorehabilitation, there are currently no criteria for prognostic evaluation, guidelines for treatment techniques, goal-assessment, and orientation of patients in the pathway of patients with central and peripheral neurological lesions and post neurotrauma. In some countries, post-acute neurorehabilitation is defined, but not by standards (intensity, material, the curriculum of education, and choice of the specialization of therapists and without defining the acute phase pathway interfaces to post-acute and long-term phase). 

One workshop group faced the challenging topic of designing a *quality improvement program for medical care in TBI*, to describe potential tools and to elaborate attainable milestones for 2025. The group presented a 5-step plan: (1) decreasing time to decision making, (2) early rehabilitation (e.g., aphasia screening, dysphagia screening, delirium screening), (3) TBI registries, (4) development of a unique and multidisciplinary TBI guideline and (5) definition of a clinical decision algorithm. For the TBI code, decreasing “door to decision making” and improving communication process (e.g., Surgery – Intensive Care Unit – Neurology) the proposed solutions consisted in the development of multidisciplinary workshops, TBI simulations, pre-hospital notification for patients with moderate/severe TBI – protocol for ambulance (notify the Emergency Room Unit, CT, neurosurgeon, neurologist), training for paramedics/nurses (correct GCS evaluation, early GCS assessment).

Another topic focused on the *medical* and *therapeutic competence* required to improve the acute, post-acute, and long-term care of TBI patients. The lack of neurosurgeons and pediatric surgeons in rural areas, the hospitalization of traumatic patients in the Internal Medicine or Neurology Ward in small hospitals, the overcrowding of the Emergency Room and the Neurosurgery Department in university centers, the poverty of specific imagistic methods, are some of the alarm signals that were triggered. Other evoked problems were: the poor collaboration between neurosurgeon – anaesthesiologist – neurologist, the deficit of neuropsychologists, long-term psychotherapists, and specialists in neurorehabilitation. In poorly developed countries, there are not enough paramedics for patient triage and not sufficient equipment in the operating units.

Potential solutions to overcome these problems would be to adopt the European and international guidelines adapted to the conditions of each country or center (national or local protocols), to develop TBI registries, to create neurotraumatology centers with multidisciplinary teams and appropriate devices, to deliver proper training of paramedics and ambulance personnel in order to recognize the severe cases and transport the patients to specialized medical services in the shortest time. The long-term strategies in order to fulfill this purpose involve cooperation in a multidisciplinary team and governmental involvement in raising funds.

The workshops also tackled the importance of multidisciplinary treatment concepts becoming the gold standard for optimal medical TBI patients’ care, discussing on how these may be achieved and with what priorities. After the debate, the group presented an optimal structure of a multidisciplinary team: *neurologists (with expertise in TBI and neurorehabilitation), neuropsychologists, physical therapists, and respiratory physiotherapists, nurses specialized in neurorehabilitation and social workers*.

In closing, the last topic challenged the role of the hosts: *What should the AMN do to support countries in their mission to improve TBI care?* Representatives asserted the need to establish a communication line and feedback cycle across all the parties involved in the patient treatment course in order to promote long-term follow-up and using novel technological solutions, such as an online TBI registry and a communication platform. This online initiative could aid in the dissemination of treatment guidelines (“*What to do? When? By whom?*”), patient pathways (“*What are the next steps?*” – decision diagram for acute, subacute, and long-term care), and neurorehabilitation guidelines. An official AMN statement on the necessity of a multidisciplinary framework across the whole course of patient treatment and follow-up was elaborated.

By the value of the debates, the quality of workshops, and prominent speakers that took part at the event, the 17th Congress of the Academy for Multidisciplinary Neurotraumatology distinguished itself as a unique opportunity to refresh one’s knowledge and to network with the world’s finest professionals in the challenging and complex field of neurotraumatology.

